# Development and Evaluation of a Novel Zwitterionic Surfactant-Based Foaming Formulation for Gas Well Deliquification

**DOI:** 10.3390/gels12100904

**Published:** 2026-10-08

**Authors:** Sanbao Dong, Yao Yan, Haifeng Liu, Jiakui Chen, Jinhua Li, Weiwei Han, Chao Ma

**Affiliations:** 1 College of Chemistry and Chemical Engineering, Xi’an Petroleum University, Xi’an 710065, China; yanyao0006@126.com (Y.Y.); chenjiakui113@126.com (J.C.); lijinhua0107@126.com (J.L.); hanweiwei@xsyu.edu.cn (W.H.); 2 Hubei Key Laboratory of Oil and Gas Drilling and Production Engineering, Yangtze University, Wuhan 430100, China; 500526@yangtzeu.edu.cn; 3 No. 2 Gas Production Plant of Yanchang Gas Field, Shaanxi Yanchang Petroleum (Group) Co., Ltd., Yulin 718500, China; liuhaifeng0910@126.com; 4 School of Petroleum Engineering, Yangtze University, Wuhan 430100, China

**Keywords:** liquid accumulation, gas well deliquification, zwitterionic surfactant, sodium alginate, foaming performance

## Abstract

A novel zwitterionic surfactant (HAPB) was synthesized and combined with lauramidopropyl trimethylammonium chloride (LATC) and alpha olefin sulfonate (AOS) to form a high-performance foaming formulation for gas well deliquification. The optimized LATC/AOS/HAPB formulation (15 mmol/L, molar ratio of 16/4/5) achieved a foaming volume and half-life of 400 mL and 9.46 min, respectively. Adding 0.2% sodium alginate (SA), a green polymer, the LATC/AOS/HAPB/SA foam’s volume dropped to 350 mL; however, its half-life ramped to 55.47 min. With 0.1 SA and 0.01% calcium lactate (CL), the LATC/AOS/HAPB/SA/CL system exhibited excellent foaming and liquid-carrying performance. At 50% methanol, its liquid-carrying rate reached its maximum (57.44%), while the foaming rate remained 100%. Condensate oil slightly reduced the LATC/AOS/HAPB/SA/CL mixture’s liquid-carrying performance, but did not affect its foaming ability, which was maintained at 100% below 50% condensate oil content. The foaming formulation demonstrated outstanding salt resistance, producing a liquid-carrying rate of 26.40% and a foaming rate of 90% at an extremely high salinity of 410 g/L, which was the highest level reported in the literature to date. Furthermore, surface tension tests and the foam’s morphological analysis verified the presence of synergy among the LATC/AOS/HAPB/SA/CL system, which effectively enhanced its foaming capability, foam stability, and liquid-carrying ability.

## 1. Introduction

Foams are widely used in oil and gas extraction and daily chemical production. The foams’ stability directly determines their application potential [1,2,3,4]. Particularly in natural gas extraction, gas well deliquification is a key method for promoting the flow and production of natural gas through gas well tubes, in which the foam serves as the working fluid. The effectiveness of foam-based deliquification is fundamentally controlled by the properties of foaming additives and foam stabilizers used [5]. High-performance foam stabilizers can significantly enhance the foam’s stability under extreme salinity and temperature conditions. Therefore, an efficient foaming formulation was developed in this study.

Foam stabilizers are mainly divided into three categories: nanomaterials, synthetic polymers, and bio-derived polymers [6]. Nanomaterials, due to their unique size effects, can play a special role in foam systems. Sun et al. combined carbon quantum dots (CQDs) and SiO_2_ nanoparticles as nano-stabilizers for a quaternary ammonium salt foaming agent [7]. This foaming system demonstrated excellent performance under simulated extreme conditions (80 °C, salinity 350 g/L) of the Hechuan Gasfield. This confirmed that the synergistic effect of CQDs and SiO_2_ nanoparticles can effectively resist the influences from high-salinity and high-oil environments. Synthetic polymers (e.g., HPAM, etc.) are widely used in oilfields due to their excellent viscosifying properties [8]. Li et al. fabricated a novel gel foaming formula containing 0.4% HPAM, 0.06% phenolic, and 0.20% betaine surfactant; its half-life was 3.8 times that of the conventional foam [9]. This increased foam stability was ascribed to the formation of the three-dimensional network within the gel foam lamella, which would increase the liquid phase’s viscosity and block the drainage process.

Besides synthesized polymers, natural polymers have started serving as foam stabilizers due to their renewable and easily degradable characteristics [10,11,12]. In the study of bio-derived polymer foam stabilizers, xanthan gum (XG) and cellulose derivatives, such as sodium carboxymethyl cellulose (NaCMC), have attracted widespread attention [13,14]. Qiu et al. developed a ternary foam drainage agent (SDBS/CHSB/XG), in which the SDBS and CHSB were used as foaming agents, while XG was used as a foam stabilizer [15]. This foaming formulation exhibited excellent foaming performance, foam stability, and liquid-carrying efficiency at high condensate oil content (up to 50%) conditions. Besides XG, sodium alginate (SA), another important biopolymer, also exhibited outstanding foam-stabilizing performance within high-temperature/salinity environments. Liu et al. investigated a composite foam system consisting of a zwitterionic surfactant (THSB), HPAM, SA and Ca^2+^; the THSB foam’s stability was contributed to by the formation of a network of the covalent crosslinked HPAM and the ionic crosslinked SA/Ca^2+^ [16]. This study highlighted the significant advantages of SA: it not only outperformed common additives like CMC in salt tolerance but also maintained stable performance at high temperatures, achieving a half-life of up to 40 days. Li et al. confirmed that 0.6% SA increased the half-life time of dodecyl dimethyl betaine (BS-12) foam to over 25 min, which is 10 times that of the pure BS-12 foam [17]. These studies indicated that SA can produce significant synergistic effects with multivalent ions and various surfactants in stabilizing foams [18]. Among these studies, the Ca^2+^ ions were released from calcium lactate (CL). Due to this controlled release of Ca^2+^ ions, the rapid local crosslinking process was prevented. A controlled crosslinking between SA and Ca^2+^ ions facilitated the formation of a robust gel network framework in situ within the thin liquid film of bubbles and the Plateau borders [19]. This network would enhance the structural elasticity and integrity of the system in high-salinity environments, effectively resisting excessive contraction. Thus, it achieves a performance breakthrough from limited salt tolerance to highly synergistic and efficient salt resistance. However, to the best of our knowledge, foams reinforced by crosslinked SA/Ca^2+^ for gas well deliquification have not been reported.

In this study, we synthesized a novel zwitterionic surfactant (HAPB), and then combined it with lauramidopropyl trimethyl ammonium chloride (LATC) and alpha olefin sulfonate (AOS) to fabricate a foaming complex. The SA and CL were used as the foam stabilizer due to the crosslinking reaction between them. After the foaming, foam-stabilizing and liquid-carrying performance evaluations, an efficient foaming formulation (LATC/AOS/HAPB/SA/CL) was ultimately optimized. This work aims to provide an efficient way to fabricating foam drainage agents for liquid unloading operation in gas wells and thus promote gas recovery in gas wells under extreme conditions.

## 2. Results and Discussion

### 2.1. FT-IR Characterization of HAPB

**Figure 1:**
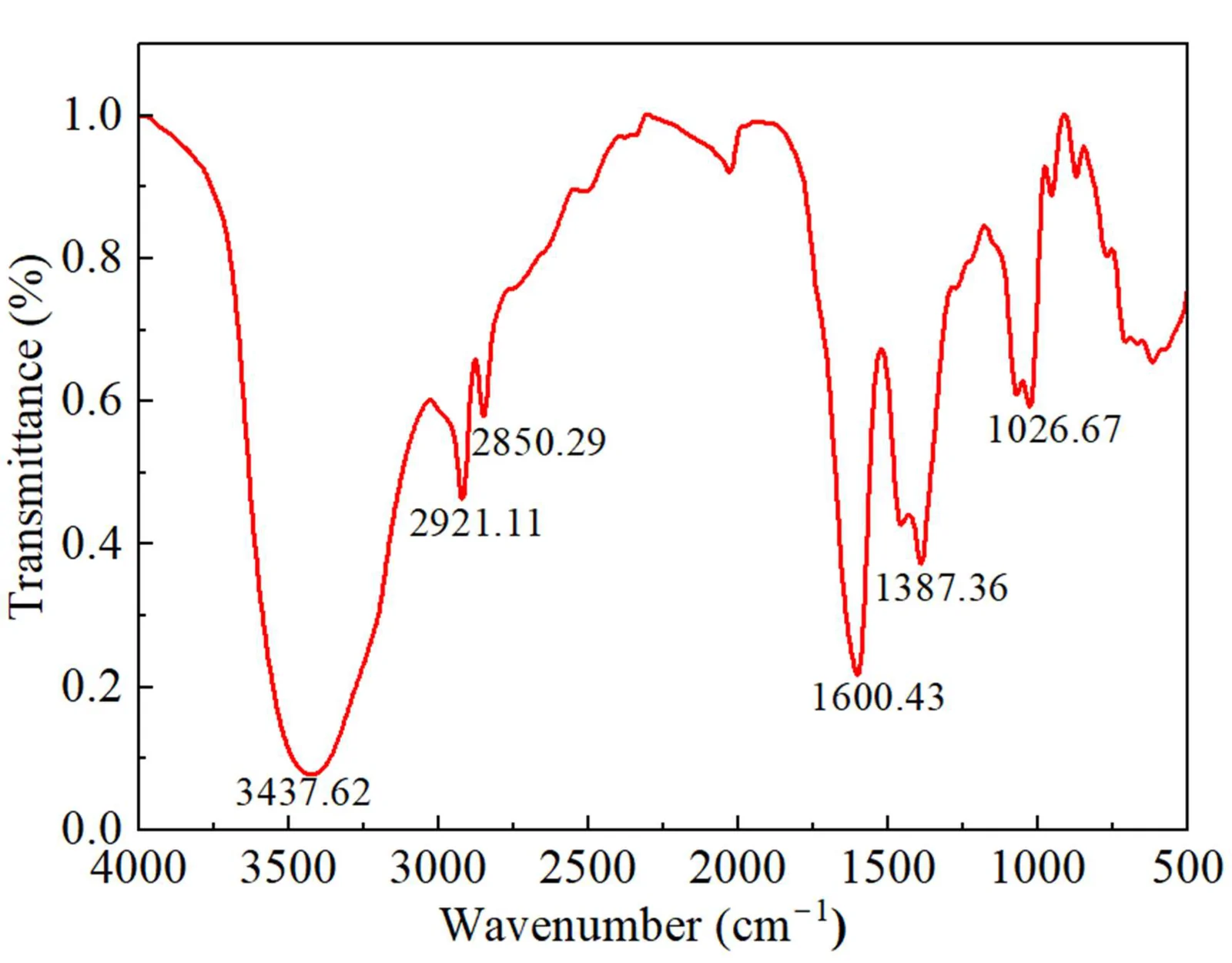
FT-IR spectrum of HAPB.

HAPB’s FT-IR spectrum is displayed in Figure 1. A strong absorption peak appears at 3437.62 cm^−1^, which corresponds to the O-H stretching. The peaks at 2921.11 cm^−1^ and 2850.29 cm^−1^ are caused by the C-H bond’s asymmetric and symmetric stretching vibrations, while the -CH_3_ groups’ bending vibration leads to the peak at 1387.36 cm^−1^. The presence of these characteristic peaks collectively confirms the existence of a saturated alkyl chain. The infrared characteristic vibrations of the -COO^−^ group can be divided into asymmetric and symmetric stretching vibrations. First, the characteristic wavenumber range for asymmetric stretching vibration is 1550–1650 cm^−1^, and the absorption peak at 1600.43 cm^−1^ in this spectrum falls within this range. Second, the characteristic wavenumber range for symmetric stretching vibration is 1350–1450 cm^−1^, and its absorption peak appears superimposed with the methyl bending vibration peak at 1387.36 cm^−1^. The presence of these two superimposed characteristic signals confirms the existence of the -COO^−^ group in the product. The characteristic wavenumber range for the C-N stretching vibration of the quaternary ammonium group (N^+^) is 900–1200 cm^−1^. In this spectrum, C-O stretching vibration produces the absorption peak at 1026.67 cm^−1^, which is also the signal of the C-N stretching vibration. This superimposed peak falls within the characteristic range for the C-N stretching vibration of the quaternary ammonium group. In summary, the characteristic absorption peaks in the infrared spectrum correspond well to the key functional groups designed in the molecular structure of the target product, including the -OH, saturated alkyl chain, -COO^−^, and -N^+^ groups. This supports the successful synthesis of HAPB [20,21].

### 2.2. Foaming and Foam-Stabilizing Capacities of Individual Surfactants

**Figure 2:**
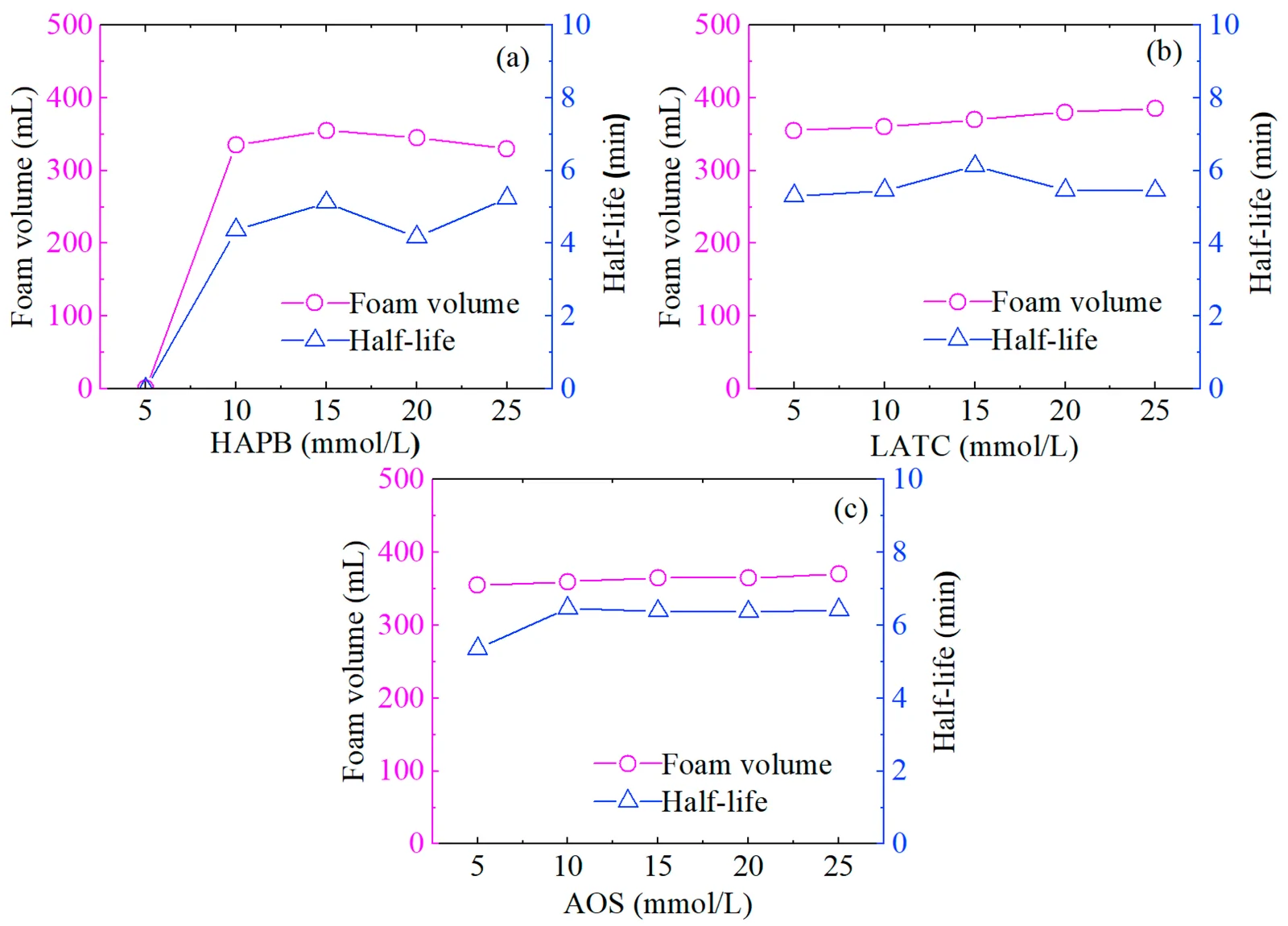
HAPB (**a**), LATC (**b**) and AOS’ (**c**) foaming and foam-stabilizing capability.

As shown in Figure 2a, HAPB exhibited an ignorable foaming ability at 5 mmol/L. The HAPB foam’s volume increased rapidly within 5–10 mmol/L and then remained relatively unchanged at approximately 345 mL at 10–25 mmol/L. The HAPB foam’s half-life ramped to its maximum value (5.24 min) at 25 mmol/L, at which foam volume was 330 mL. Figure 2b exhibits that the foam volume of LATC rose from 355 mL to 385 mL within the region of 5–25 mmol/L, while the foam half-life produced a maximum value of 6.12 min at 15 mmol/L. Figure 2c indicates that the foam volume of AOS remained relatively stable, ranging between 355 mL and 370 mL, within the 5–25 mmol/L concentration interval, and the maximum half-life (6.47 min) appeared at 10 mmol/L and displayed a generally flat trend overall. These experimental results indicate that the single components’ foaming and foam-stabilizing performance needed to be further enhanced. To achieve comprehensive optimization of foam performance, a compounding strategy was adopted in this study for system improvement.

### 2.3. Foaming and Foam-Stabilizing Properties of LATC/AOS Mixture

**Figure 3:**
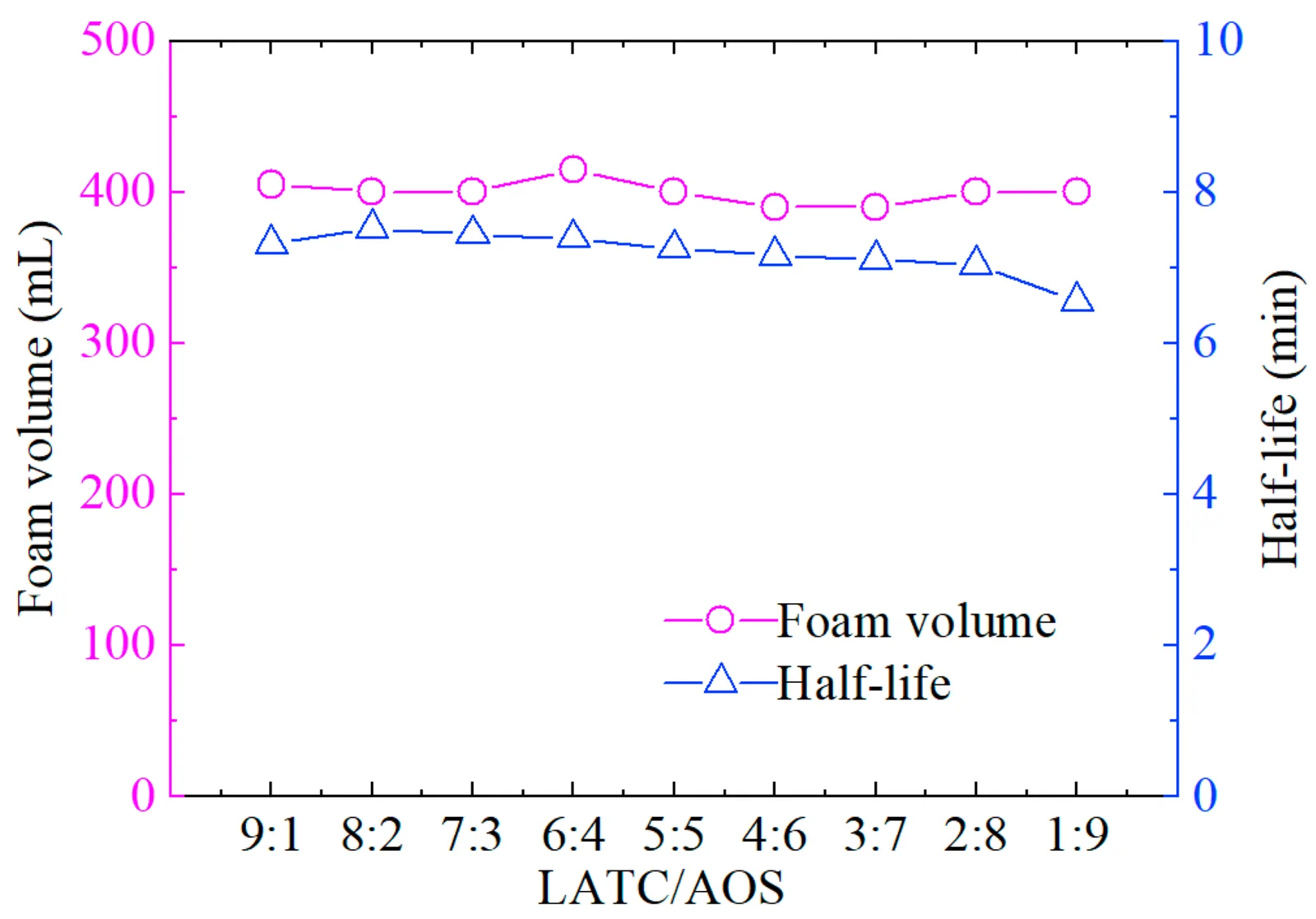
Foaming volumes and half-lives at various LATC/AOS molar ratios.

Subsequently, the foaming performance of binary mixtures of LATC and AOS at different mixing ratios was investigated, with the total surfactant concentration maintained at 15 mmol/L for each measurement. As plotted in Figure 3, the maximum foaming volume (415 mL) appeared when the LATC/AOS molar ratio met 6:4, where the corresponding half-life was 7.39 min. Regarding the trend in half-life, its value first increased and then decreased with varying mixing ratios, reaching a peak of 7.52 min at a molar ratio of 8:2, at which point the foam volume climbed up to 400 mL. Compared to the individual components (LATC and AOS), both the foam volume and half-life were vastly improved. Considering the purpose of fabricating a foaming mixture with both high foaming volume and foam-stabilizing ability, the LATC/AOS molar ratio was adopted as 8:2 for optimizing the ternary foaming mixtures.

### 2.4. Optimization of LATC/AOS/HPAB Mixture

**Figure 4:**
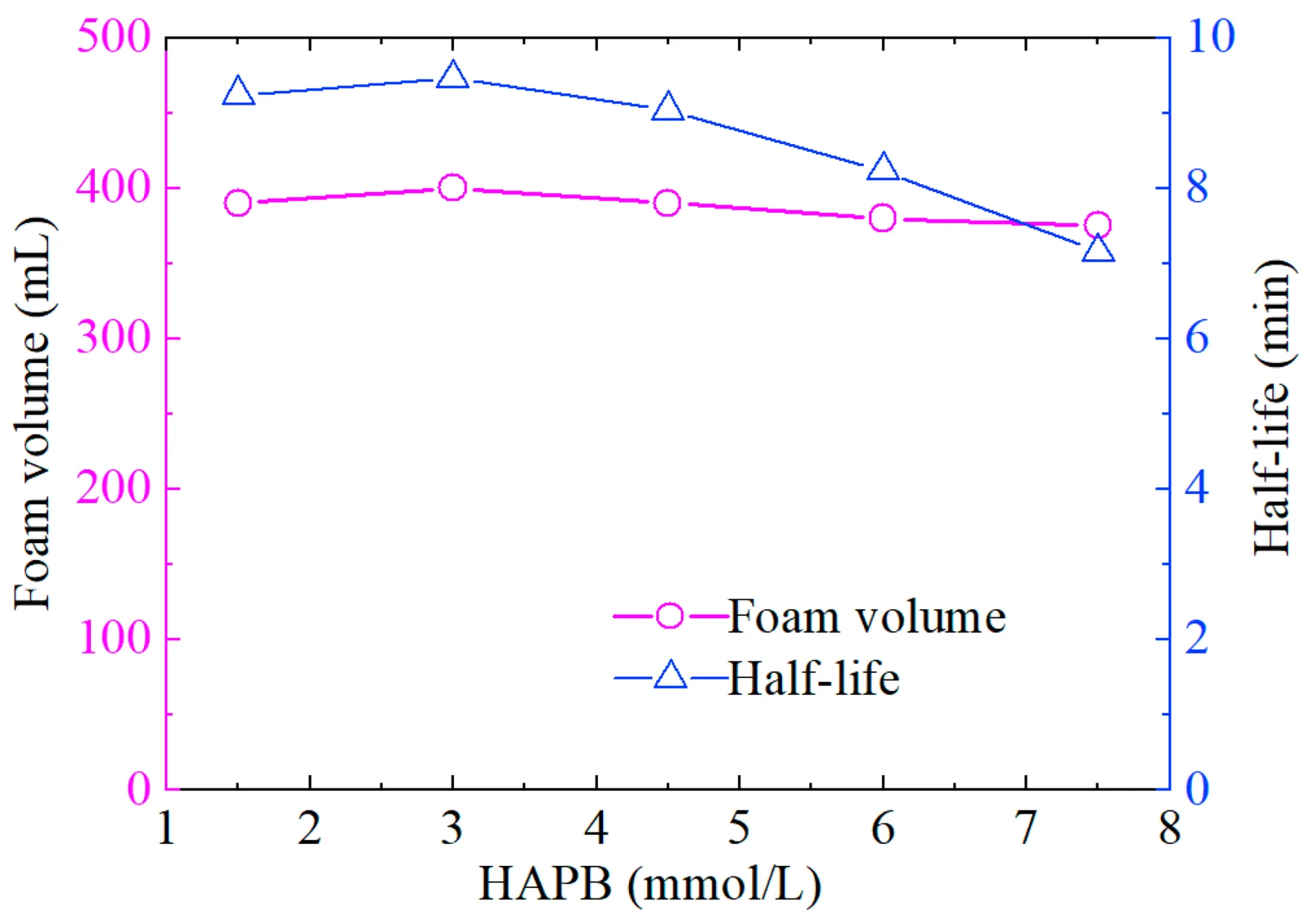
The effect of HAPB on the properties of LATC/AOS/HAPB.

In this section, the synthesized HAPB was incorporated into the LATC/AOS system as a third component to see if a higher foaming and foam-stabilizing performance could be generated. The ratio of LATC/AOS was fixed at 8:2, and the total concentration of LATC/AOS/HAPB was maintained at 15 mmol/L. The foam volume and half-life profiles were presented in Figure 4, from which it could be found that with the addition of HAPB, the maximum foam volume of 400 mL was achieved at an HAPB dosage of 3 mmol/L, at which the half-life also reached its maximum value (9.46 min). As the HAPB dosage ranged from 3 to 7.5 mmol/L, both the foam volume and half-life dropped gradually, which were from 400 mL to 375 mL, and from 9.46 min to 7.15 min, respectively. Herein, the optimized HAPB dosage in the ternary mixture LATC/AOS/HAPB was 3 mmol/L, the corresponding optimal molar ratio of LATC/AOS/HAPB was 16:4:5.

**Figure 5:**
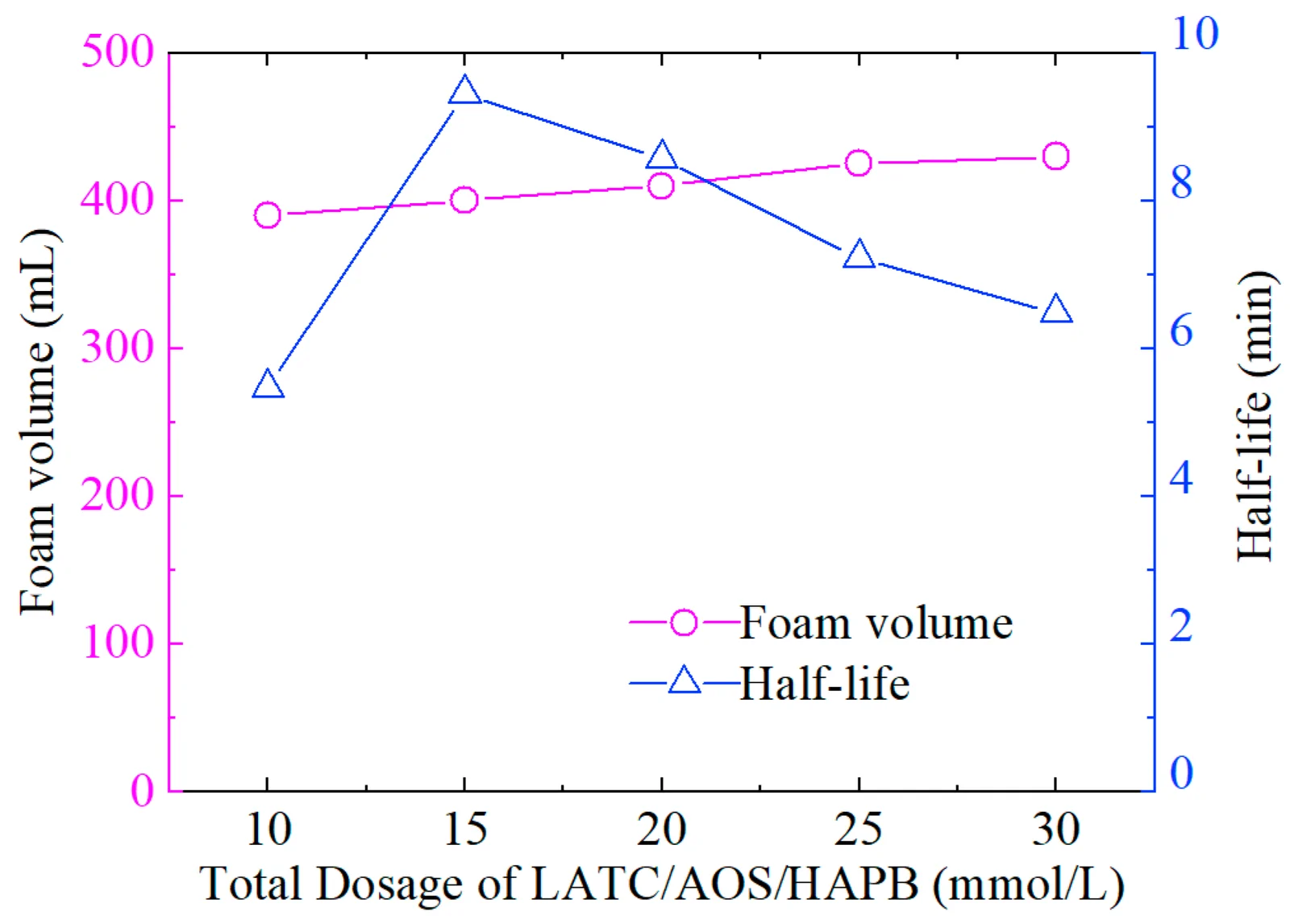
The total dosage of LATC/AOS/HAPB (16:4:5) on its foaming and foam-stabilizing properties.

Finally, as shown in Figure 5, the total dosage of LATC/AOS/HAPB posed a significant influence on the foaming and foam-stabilizing performance. As indicated by Figure 5, the foam volume of LATC/AOS/HAPB increased from 390 mL at a total concentration of 10 mmol/L to 430 mL at 30 mmol/L. The half-life, however, appeared to show an initial increase and then a decrease, producing a maximum of 9.46 min at 15 mmol/L, with a corresponding foam volume of 400 mL. Therefore, the optimal total concentration for the ternary mixture system LATC/AOS/HAPB turned out to be 15 mmol/L. This result also demonstrated that the compounding of LATC, AOS, and HAPB was a superior method to producing a high-performance foaming formulation.

### 2.5. Effect of SA Dosage on Foaming Properties and Foam Stability

**Figure 6:**
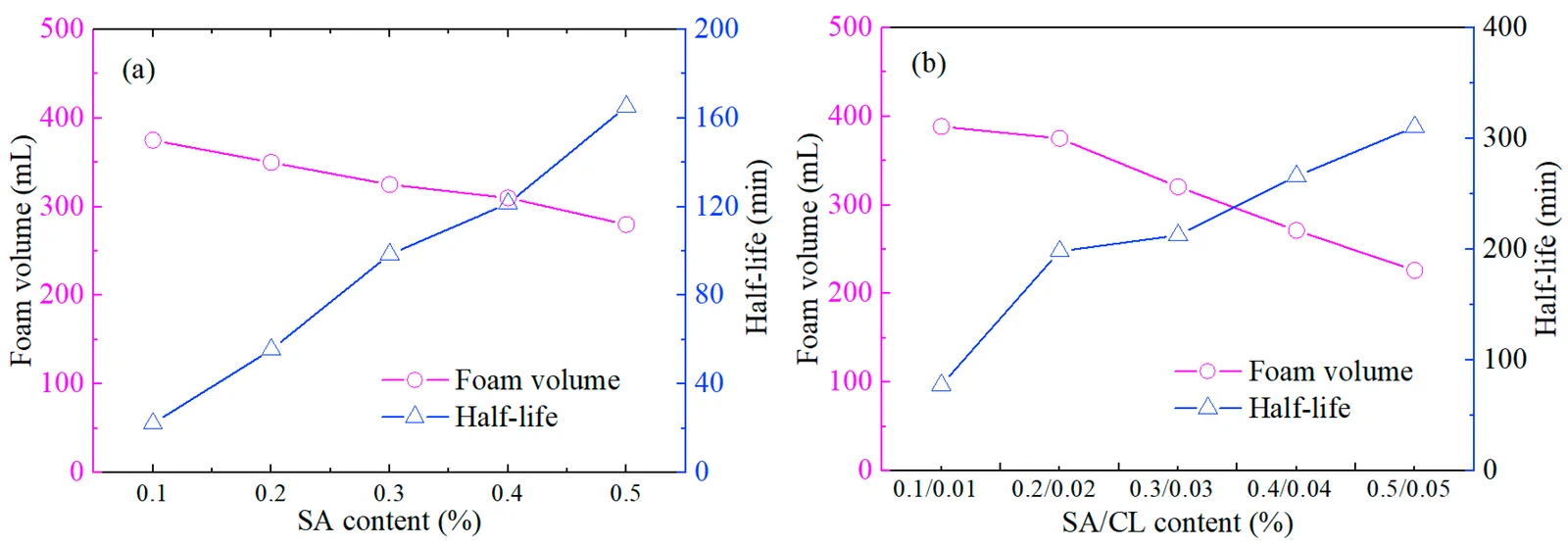
(**a**) SA and (**b**) SA/CL dosage’s effect on the LATC/AOS/HAPB’s properties (15 mmol/L, with a molar ratio of 16:4:5).

With the presence of SA, a naturally derived anionic polysaccharide, a three-dimensional network structure in the aqueous environment could form through its molecular chains, significantly enhancing the bulk liquid viscosity [22,23], which would slow down the foam’s liquid drainage [24], thereby delaying the foam rupture process. In this section, we investigate the SA dosage’s influence on the foaming and foam-stabilizing performance of the ternary LATC/AOS/HAPB mixtures (15 mmol/L and a molar ratio of 16:4:5). As displayed in Figure 6a, the addition of SA could significantly increase the LATC/AOS/HAPB’s foam-stabilizing performance, while slightly retarded the foaming ability. The half-life reached approximately 165.23 min at an SA content of 0.5%. Conversely, the foam volume curve appeared as a gradually decreasing trend, from 375 mL at 0.1% SA to 280 mL at 0.5% SA. Due to the balance between the stability and flowability of the SA-reinforced foam, the proper dosage of SA was selected as 0.2%. From Figure 6b, with the presence of SA/CL (at a concentration ratio of 10:1), it was found that the LATC/AOS/HAPB/SA/CL’s foam-stabilizing performance was much higher than that of the LATC/AOS/HAPB complex. With the increase of SA/CL from 0.1%/0.01% to 0.5%/0.05%, the LATC/AOS/HAPB/SA/CL foam’s half-life ramped from 77.46 min to 310.39 min. However, due to the balance between the stability and flowability of the SA-reinforced foam and the SA/CL-reinforced foam, the proper dosage of SA and SA/CL were selected as 0.2% and 0.1%/0.01%, respectively.

### 2.6. Foaming and Liquid-Carrying Properties Under Different Conditions

**Figure 7:**
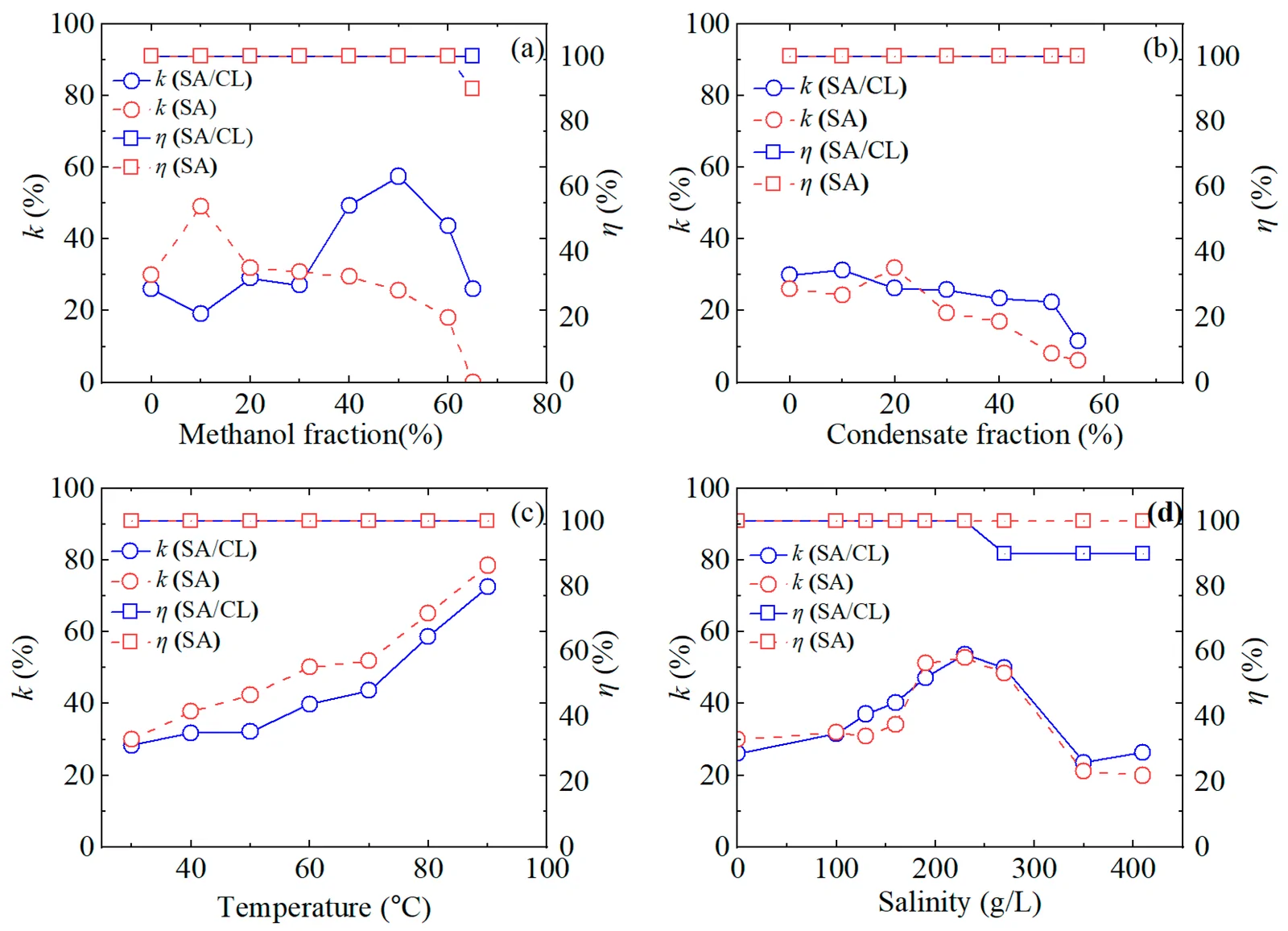
Effect of (**a**) methanol, (**b**) condensate oil, (**c**) temperature and (**d**) salinity on the properties of LATC/AOS/HAPB/SA/CL (denoted as SA/CL) and LATC/AOS/HAPB/SA (denoted as SA).

The influences of methanol, condensate, temperature, and salinity on the foaming and liquid-carrying properties of LATC/AOS/HAPB (15 mmol/L, with a molar ratio of 16:4:5) with the presence of SA (at a dosage of 0.2%) or SA/CL (at a dosage of 0.1%/0.01%) were investigated, the results of which are presented in Figure 7.

Figure 7a demonstrates the variation patterns of the liquid-carrying and foaming properties for the LATC/AOS/HAPB-blend mixture under different methanol contents. As shown in Figure 7a, low methanol content positively enhanced the LATC/AOS/HAPB’s performance: with 0.2% SA and 10% methanol, the LATC/AOS/HAPB mixture achieved a peak liquid-carrying efficiency of 49.11%. Although the liquid-carrying efficiency showed a declining trend as methanol content increased from 20% to 40%, it remained higher than that without methanol. When the methanol content increased to the region of 50–60%, the liquid-carrying efficiency decreased significantly (even lower than that of the LATC/AOS/HAPB/SA). It was obvious that methanol content had a negligible impact on the foaming efficiency (*η*), which was steady at 100%. With the presence of SA/CL, the maximum liquid-carrying efficiency (57.44%) appeared at a methanol content of 50%, at which the foaming efficiency was 100%. With 10–30% methanol, LATC/AOS/HAPB/SA/CL exhibited relatively lower liquid-carrying efficiency than that of LATC/AOS/HAPB/SA. However, at methanol contents higher than 30%, the liquid-carrying efficiency of LATC/AOS/HAPB/SA/CL was much higher than that of LATC/AOS/HAPB/SA. This indicated that methanol’s performance-enhancing effect is more pronounced for the LATC/AOS/HAPB/SA/CL mixture, which might be ascribed to the formation of SA/CL networks that increase the aqueous solution phase and inhibit the penetration of methanol molecules through the foam liquid film. The penetration of gas molecules usually accelerates the collapse of the foam [25].

The liquid-carrying and foaming performance of LATC/AOS/HAPB/SA and LATC/AOS/HAPB/SA/CL under different condensate oil contents are shown in Figure 7b. For the LATC/AOS/HAPB/SA mixture, with 10% condensate oil, the maximum liquid-carrying efficiency (31.34%) appeared, indicating that low condensate oil content slightly enhanced the system’s liquid-carrying performance. As the condensate oil content increased to 20–55%, the liquid-carrying efficiency decreased from 26.29% to 11.56%, during which the foaming efficiency was steady at 100%. For the LATC/AOS/HAPB/SA/CL mixture, although the overall liquid-carrying efficiency was generally higher than that generated by the LATC/AOS/HAPB/SA mixture, it consistently exceeded the latter when the condensate oil content was from 0% to 55%, confirming that the introduction of CL effectively improved the system’s resistance to condensate oil. The general negative effect of condensate on the foaming and liquid-carrying properties might be ascribed to the spontaneous spreading nature of condensate on the water–air interface, thus exhibiting a defoaming effect [26]. Contrary to our justification, the presence of a low content of condensate oil exhibited as a weak foam stabilizer, the reason for which was hard to reveal. However, in some publications, similar phenomena were also observed by other researchers [27,28].

The temperature’s effects on the performance of LATC/AOS/HAPB/SA and LATC/AOS/HAPB/SA/CL are presented in Figure 7c. For the LATC/AOS/HAPB/SA mixture, the liquid-carrying efficiency showed a nearly linear increase within the tested temperature range (30 °C to 90 °C). The liquid-carrying efficiency was 29.95% at room temperature (30 °C) and reached 78.53% at 90 °C. This verified that increasing temperature enhanced the system’s liquid-carrying performance, and the surfactant molecules maintained good resistance to high temperatures. Throughout the entire temperature range, the LATC/AOS/HAPB/SA mixture’s foaming efficiency remained at 100%, suggesting that temperature (up to 90 °C) had no negative effect on foaming performance. For the LATC/AOS/HAPB/SA/CL mixture, the liquid-carrying efficiency also increased linearly with temperature, but its overall liquid-carrying capacity was slightly lower than that of the LATC/AOS/HAPB/SA mixture. The LATC/AOS/HAPB/SA mixture’s resistance to temperature was probably due to the formation of SA/CL networks that increase the aqueous solution phase, even at high temperatures up to 90 °C [29].

To check the salinity’s influence on the properties of LATC/AOS/HAPB/SA and LATC/AOS/HAPB/SA/CL, simulated formation water with different salinities composed of NaCl and CaCl_2_ (with a mass ratio of 4:1) was used. The results are presented in Figure 7d. For the LATC/AOS/HAPB/SA mixture, the liquid-carrying efficiency was 31.96% at a 100 g/L salinity, which exceeded the 29.95% observed at 0 g/L salinity. Within the salinity range of 100–230 g/L, the liquid-carrying efficiency increased with rising salinity, reaching a peak of 52.75% at 230 g/L. When the salinity exceeded 230 g/L, the liquid-carrying efficiency gradually decreased with further increases in salinity. Notably, within the 10–320 g/L salinity range, the LATC/AOS/HAPB/SA mixture’s foaming efficiency was steady at 100%, indicating that salinity has no obvious impact on foaming performance. For the LATC/AOS/HAPB/SA/CL mixture, the liquid-carrying efficiency was slightly lower than that produced by the LATC/AOS/HAPB/SA blend within the 100–160 g/L salinity range. However, in the 190–410 g/L salinity range, the LATC/AOS/HAPB/SA/CL mixture generated slightly higher liquid-carrying efficiency than that produced by the LATC/AOS/HAPB/SA mixture. At 410 g/L salinity, the liquid-carrying efficiency of the LATC/AOS/HAPB/SA/CL mixture was 26.40%, which might be the highest salt resistance of foaming fluids for gas well deliquification reported. The high salt resistance of the foaming LATC/AOS/HAPB/SA/CL mixture might be due to the presence of HAPB and SA molecules, in which the HAPB was a zwitterionic surfactant with a good salt resistance nature and the SA molecules had a large amount of carboxylate groups that chelated the Ca^2+^ ions in the aqueous phase [30].

### 2.7. Synergistic Effects in the LATC/AOS/HAPB Mixture

**Figure 8:**
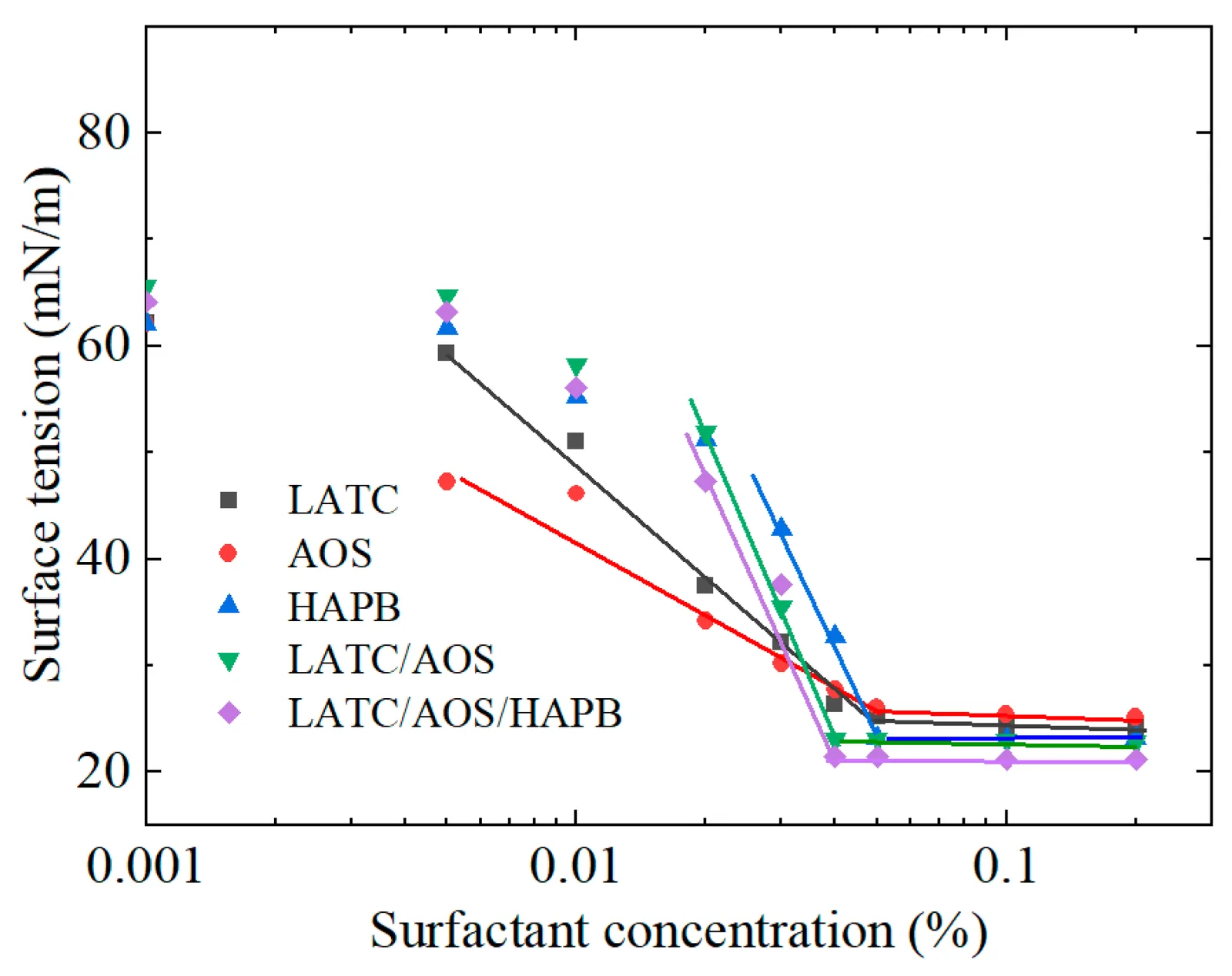
Surfactant solutions’ surface tensions at room temperature.

Low surface tension usually poses as a key factor favoring foam generation and foam stabilization [31,32]. It should be noted that an aqueous solution with lower surface tension [33] generally has higher foamability, while low surface tension alone is not enough to produce a foam with high stability [34]. Figure 8 displays the surface tension plots for solutions with single surfactants (LATC, AOS, and HAPB), binary mixtures (LATC/AOS with molar ratio 8/2), and ternary mixtures (LATC/AOS/HAPB with molar ratio 16/4/5). It can be observed that each curve decreased linearly with increasing concentration and then kept unchanged after a specific point (CMC) [35]. Compared to the binary LATC/AOS system and the ternary LATC/AOS/HAPB surfactant mixture, the single-surfactant HAPB exhibited the highest CMC at 0.05%, while the CMC values for both the LATC/AOS and LATC/AOS/HAPB mixtures were ~0.04%. As clearly shown in Figure 8, the equilibrium surface tensions of the LATC/AOS and LATC/AOS/HAPB mixtures were 22.87 mN/m and 21.17 mN/m, respectively, which dropped vastly compared to those produced by AOS (25.11 mN/m) and LATC (24.22 mN/m). This indicates that the blending of certain types of surfactants can reduce the equilibrium surface tension to a lower value, which facilitates the foaming properties of a foaming solution. In this section, the LATC/AOS/HAPB mixture generated the lowest surface tension, which means that the LATC/AOS/HAPB solution had the highest foaming ability, consistent with data obtained in the foaming-capacity and foam-stabilizing tests.

**Figure 9:**
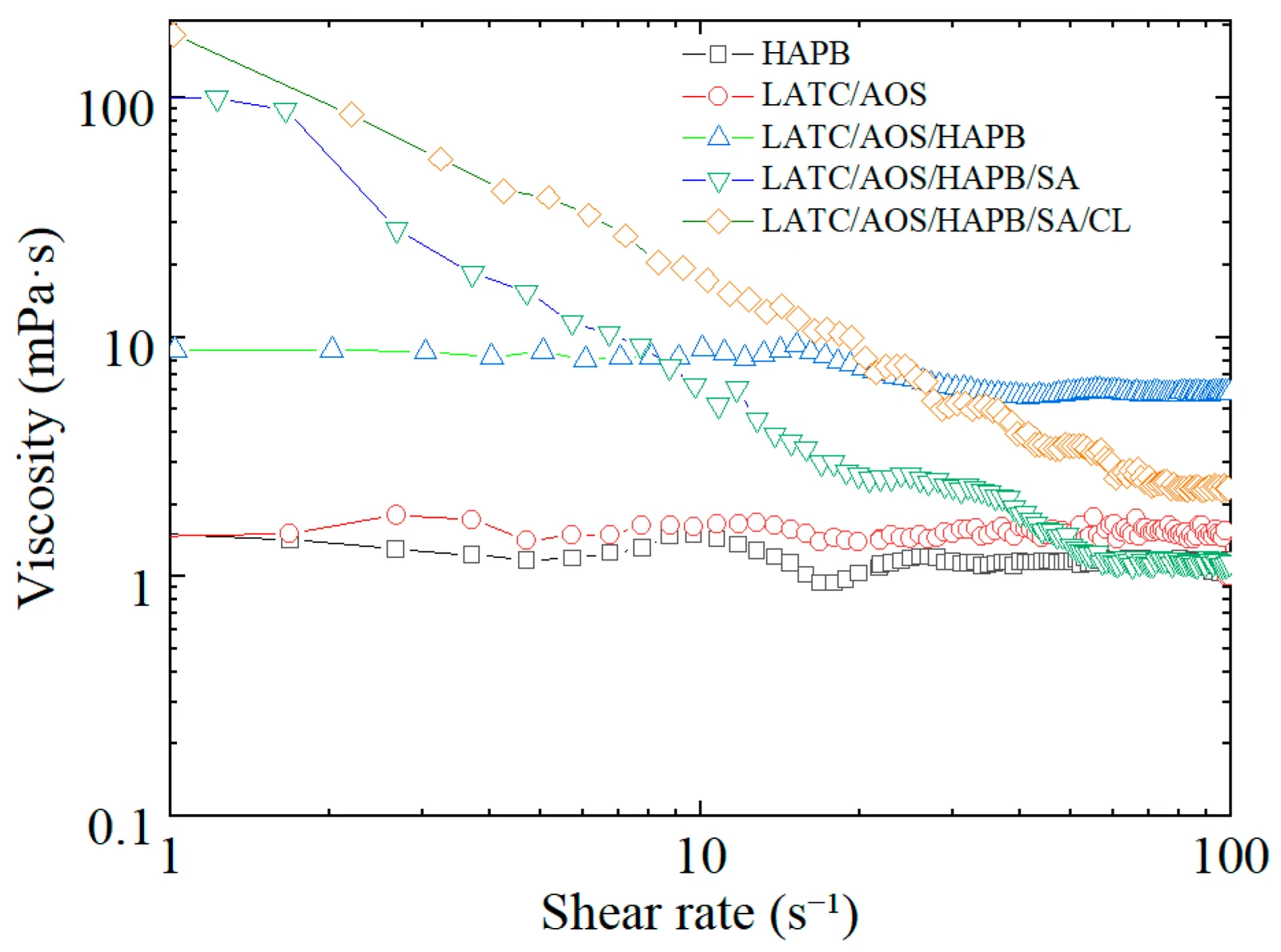
Apparent viscosities of solutions at shearing and room temperature conditions (LATC/AOS ratio is 4:1, and LATC/AOS/HAPB ratio is 16:4:5, each with a total dosage of 15 mmol/L).

**Figure 10:**
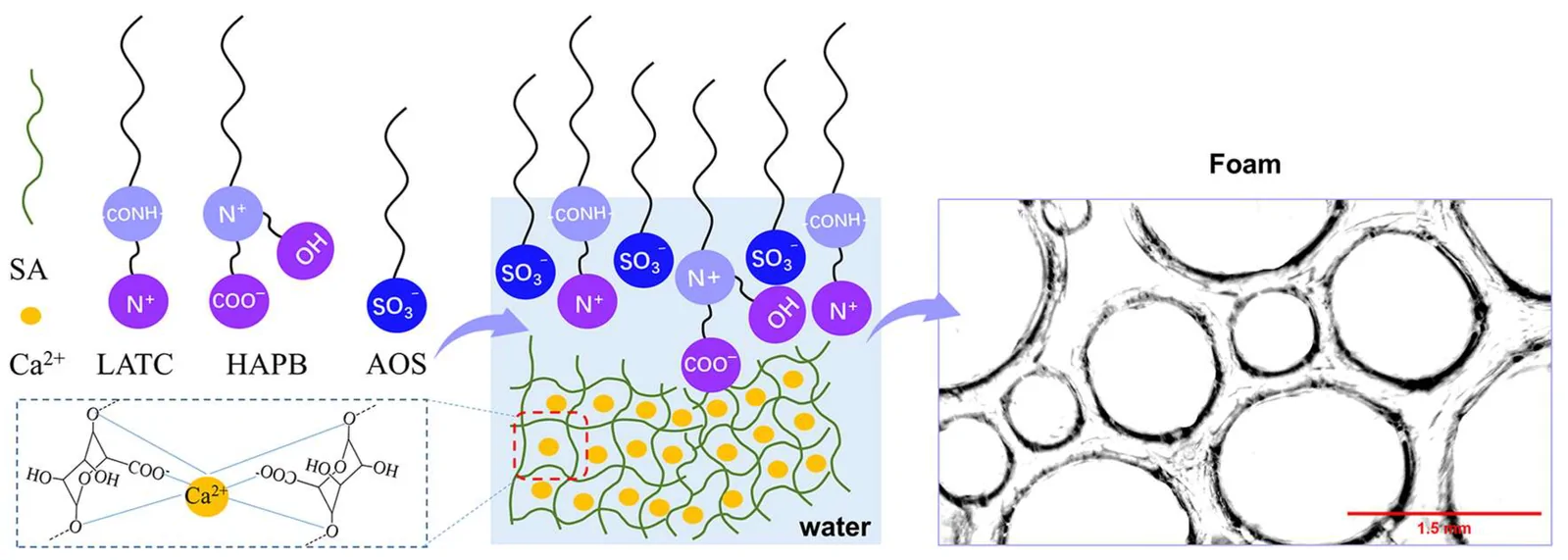
The possible mechanism of SA/CL in favoring the foam’s stability.

Figure 9 shows the apparent viscosities of various solutions under shearing conditions (1–100 s^−1^). The viscosity of the 15 mmol/L HAPB solution remained almost constant within 1 to 100 s^−1^, maintaining values around 1.2 mPa·s, exhibiting a Newtonian fluid behavior [36]. The viscosity of the LATC/AOS solution remained around 1.4 mPa·s, while the solution with the LATC/AOS/HAPB mixture gradually decreased from 8.8 mPa·s to 5.7 mPa·s. Figure 9 also clearly shows that the viscosity of the LATC/AOS/HAPB solution was higher than that of the HAPB solution and LATC/AOS solution, which would also enhance the LATC/AOS/HAPB foam’s stability. With the addition of 0.2% SA, the maximum viscosity of the LATC/AOS/HAPB/SA solution reached 99.0 mPa·s, exhibiting shear-thinning behavior, and finally decreasing to 1.2 mPa·s. With the addition of SA/CL (0.1%/0.01%) for testing, Figure 9 reveals that the maximum viscosity of the LATC/AOS/HAPB/SA/CL solution reached 189.0 mPa·s, also showing a shear-thinning behavior, with the viscosity finally decreasing to 2.3 mPa·s at 100 s^−1^. This shear-thinning behavior was likely due to the formation of wormlike micelles and networks under weak shearing conditions [37], while the micellar and network structures might also be broken down under higher shearing conditions, leading to the observed decrease in viscosity [38]. The networks were formed through the ionic crosslinking interaction between Ca^2+^ (from CL molecules) and -COO^−^ groups from SA, as sketched in Figure 10. The viscous nature of the LATC/AOS/HAPB/SA/CL solution would restrain the gravity drainage phenomenon of the LATC/AOS/HAPB/SA/CL foam and reasonably improve the foam’s liquid-carrying performance.

As described in Figure 10, at the air–water interface, the LATC, AOS and HAPB molecules accumulated due to their amphiphilic properties. In addition, the electrostatic attractions and hydrogen bonds among them drove them to form a closely packed adsorption layer, thereby enhancing the stability of the films of the foam. Furthermore, the SA and CL molecules mostly stayed in the bulk water phase, and the ionic crosslinking interaction occurred between Ca^2+^ ions (from CL molecules) and -COO^−^ (groups from SA); herein, the viscosity of the bulk water phase could be strongly enhanced, as confirmed in Figure 9, which also contributed to the stability of the foams.

### 2.8. Results of Quantum Chemical Computation

**Figure 11:**
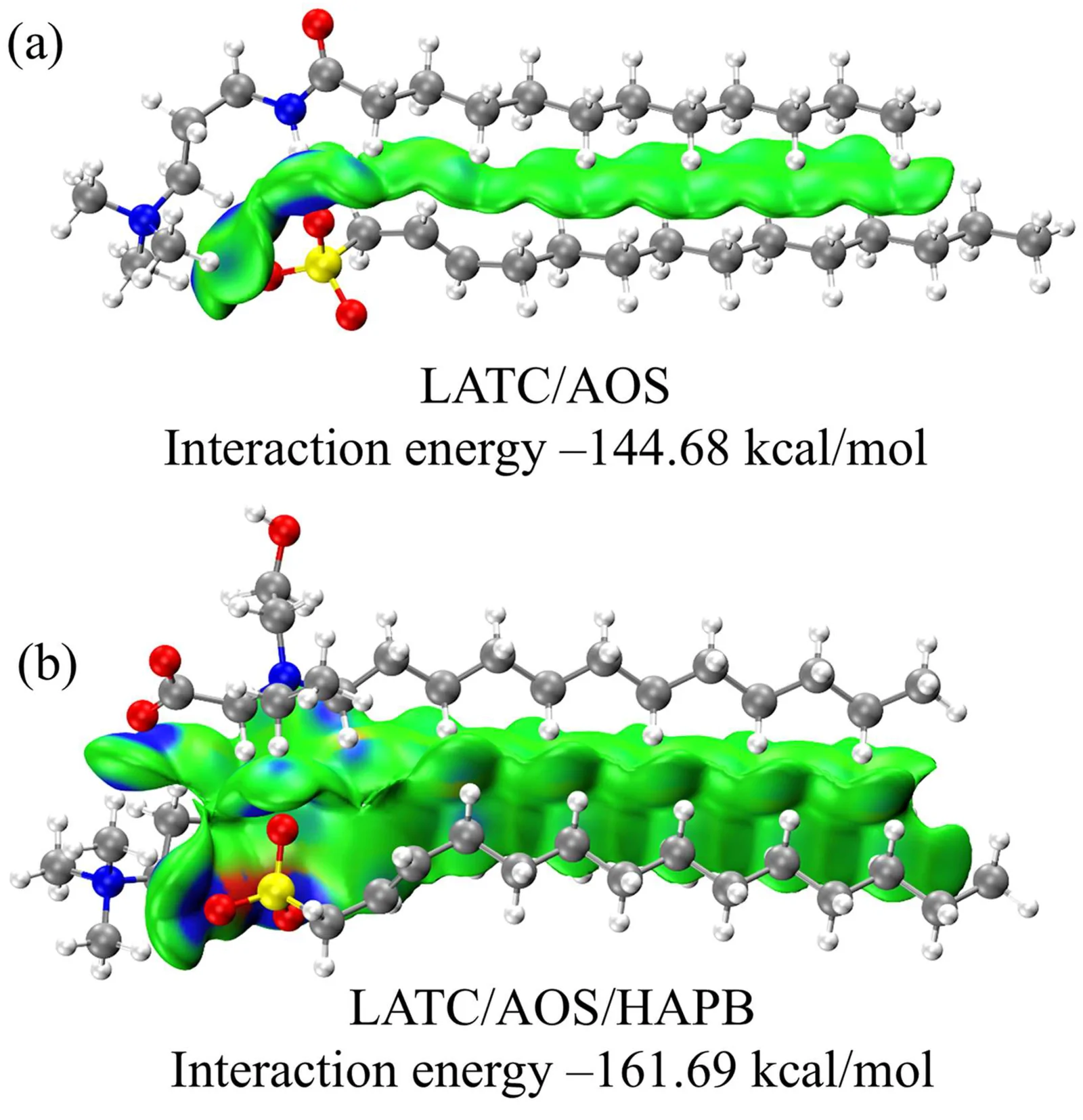
The interaction energies within the complexes of LATC/AOS (**a**) and LATC/AOS/HAPB (**b**).

To get a deeper insight into the supramolecular interactions (e.g., electrostatic attraction, hydrogen bonding, etc.) within the complexes of LATC/AOS and LATC/AOS/HAPB, we conducted quantum chemical calculations using xtb software (version 6.4.1). As depicted in Figure 11a,b, the interaction energy between LATC and AOS was −144.68 kcal/mol, while that among LATC, AOS and HAPB turned out to be −161.69 kcal/mol, which meant that when the LATC, AOS and HAPB were present in the solution, a closely packed adsorption layer on the foam’s films could be formed. Therefore, a lower surface tension produced by LATC/AOS/HAPB could be realized (as verified in Figure 8), which would facilitate the foaming agent’s foaming ability and foam-stabilizing performance, as is confirmed in Figure 2, Figure 3, Figure 4 and Figure 5.

**Figure 12:**
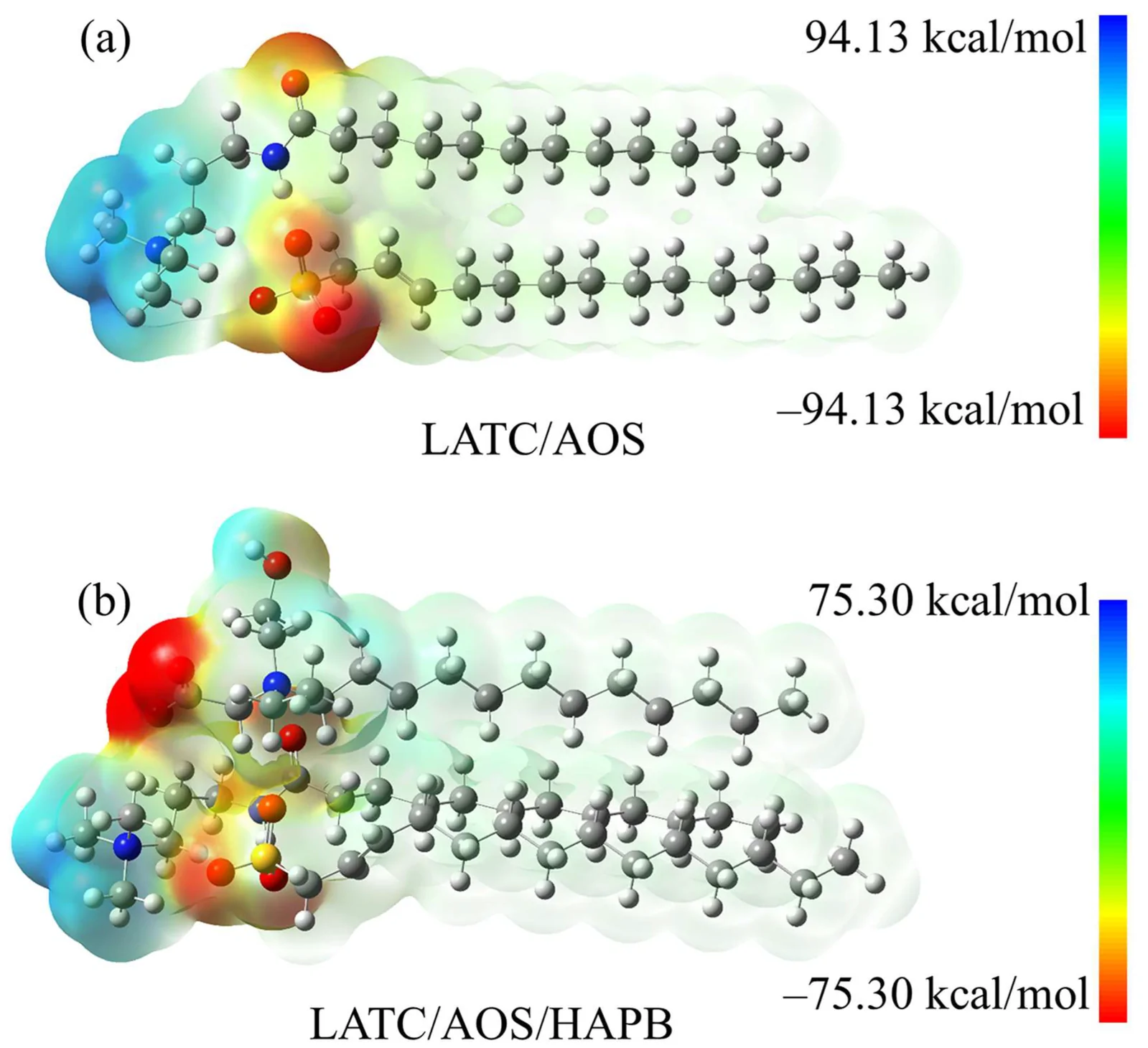
The electrostatic surface potentials of LATC/AOS (**a**) and LATC/AOS/HAPB (**b**).

In addition to the binding energy calculations, we have also the electrostatic surface potentials of LATC/AOS and LATC/AOS/HAPB, depicted in Figure 12. In Figure 12a, it is obvious that there is a big difference in electrostatic surface potential between the hydrophilic head of LATC and the hydrophilic head of AOS. As an anionic surfactant, the hydrophilic group was the -SO_3_^−^ group, showing an electronegative nature, while as a cationic surfactant (LATC), its quaternary ammonium group showed an electropositive nature, meaning that the presence of a strong electrostatic attraction between them, herein the combination of LATC and AOS would reasonably produce a synergistic effect in enhancing the foaming and foam-stabilizing ability. A similar phenomenon is also present in Figure 12b; the obvious differences among the colors of the hydrophilic groups of LATC, AOS and HAPB also mean that the compounding of them would also result in a strong electrostatic attraction, which would induce the enhancement of the solution’s foaming and foam-stabilizing ability, which has been proved previously in this study.

### 2.9. Microscopic Morphology Analysis of Foam

**Figure 13:**
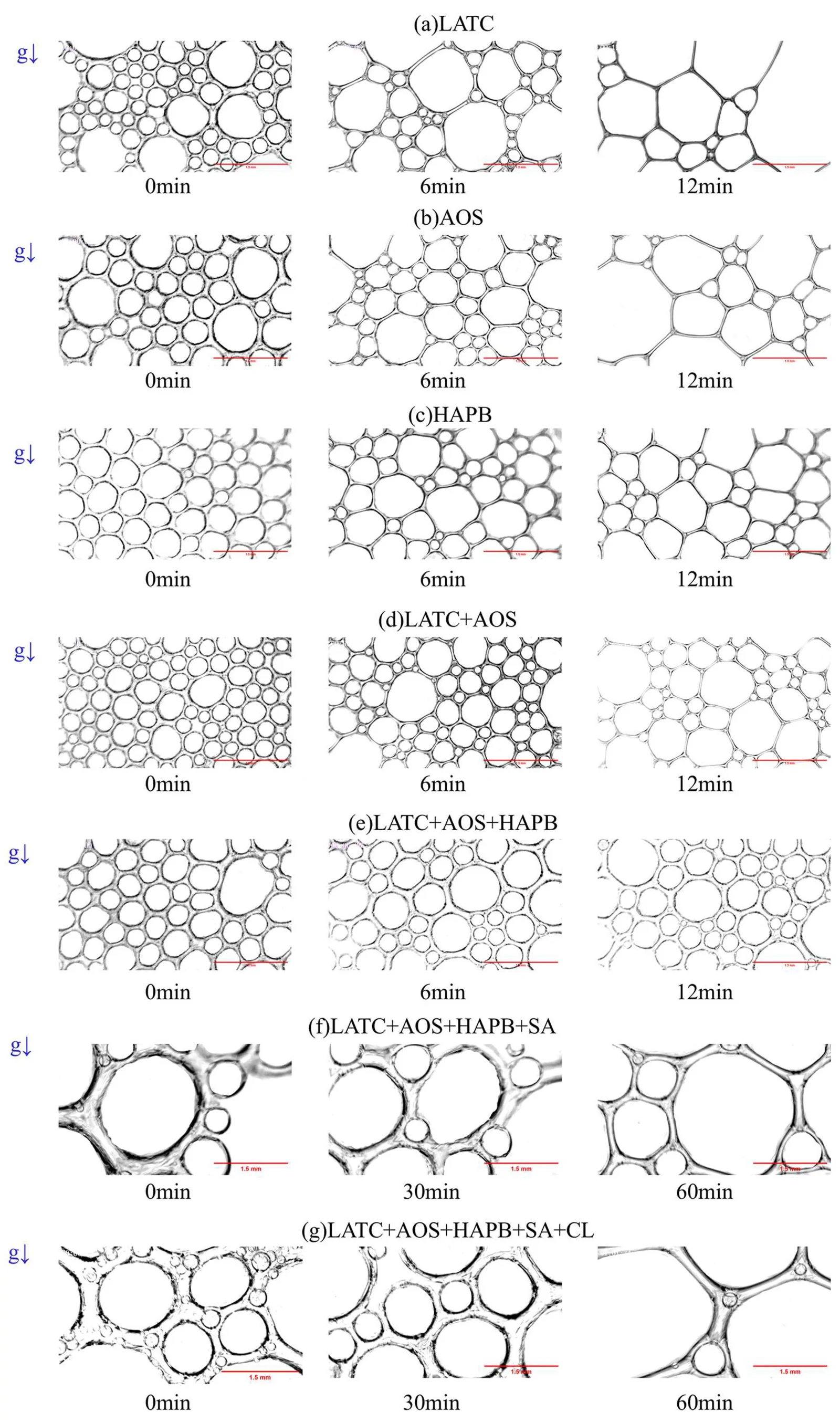
Foam evolution process of each foam stabilized by (**a**) LATC, (**b**) AOS, (**c**) HAPB, (**d**) LATC/AOS, (**e**) LATC/AOS/HAPB, (**f**) LATC/AOS/HAPB/SA, (**g**) LATC/AOS/HAPB/SA/CL, the scale is 1.5 mm, each arrow (**g**) represents the gravity.

Figure 13 shows the microscopic foam structures of different surfactant solutions, each of which contained a surfactant concentration of 15 mmol/L. The foam’s film thickness was used as an effective indicator for evaluating the foam’s liquid-carrying capacity. Figure 13 displays the changes in foams with different surfactants. Figure 13a–c correspond to the foam morphologies of the foams stabilized by LATC, AOS, and HAPB, respectively. Figure 13d and Figure 13e represent the morphological changes of LATC/AOS foam (molar ratio 4:1) and LATC/AOS/HAPB foam (molar ratio 16/4/5), respectively. Figure 13f represents the LATC/AOS/HAPB/SA foam, in which the SA content was 0.2% and the LATC/AOS/HAPB molar ratio was 16/4//5. Figure 13g represents the LATC/AOS/HAPB/SA/CL foam, in which the SA and CL contents were 0.2% and 0.02%, and the LATC/AOS/HAPB molar ratio was 16/4/5. The total surfactant concentration in each foam was fixed at 15 mmol/L.

At the initial moment (0 min), the liquid film thickness of the foams with single surfactants followed the order LATC (121.24 μm) > AOS (110.11 μm) > HAPB (108.02 μm). The values were close to each other and all fell within the range of 100–125 μm. As time extended to 12 min, the liquid film thicknesses of the foams with LATC, AOS and HAPB dropped to 28.34 μm, 33.03 μm and 33.68 μm, respectively. Such significant reduction in each film’s thickness would lead to rapid foam rupture, confirming that a single surfactant was not enough to stabilize the foams.

The foam with LATC/AOS exhibited an initial liquid film thickness of 124.79 μm, slightly higher than that of the single LATC system. After 12 min, the thickness maintained at 40.34 μm, indicating a noticeably slower thinning rate compared to each foam with a single surfactant. Remarkably, the foam with LATC/AOS/HAPB produced an initial film thickness of 156.61 μm, which was much higher than that produced by LATC (121.24 μm). Furthermore, after 12 min, the film thickness of the LATC/AOS/HAPB foam still retained a thickness of 127.82 μm, which was higher than that produced by the single-surfactant foams or binary-surfactant foams.

In this study, the polymer SA was introduced as a foam-stabilizing additive. The results revealed that with the addition of 0.2% SA, the liquid film thickness of the LATC/AOS/HAPB/SA foam turned out to be 189.44 μm. Moreover, after 60 min, the film thickness still maintained at 93.24 μm, which meant that the foam drainage process had been significantly slowed. Furthermore, with the addition of SA/CL (0.1%/0.01%) to the LATC/AOS/HAPB complex (15 mmol/L, with a molar ratio of 16:4:5), the LATC/AOS/HAPB/SA/CL foam produced an initial film thickness of 216.01 μm. Remarkably, even after 60 min, the film thickness remained at 139.17 μm, which was the highest value obtained in this study. This high stability might be caused by the crosslinking effect among CL and SA via the bonding between Ca^2+^ (from CL) and the -COO^−^ from the SA molecular chains, constructing a three-dimensional network, and thereby increasing the bulk liquid phase’s viscosity and enhancing the foam system’s stability. Therefore, the compounding of SA and CL to the foaming component (LATC/AOS/HAPB) provided a choice for building an efficient foam drainage agent for gas well deliquification [18].

## 3. Conclusions

In this study, we developed a compounded foam drainage agent LATC/AOS/HAPB, in which the recommended molar ratio and total dosage of LATC/AOS/HAPB was 16/4/5 and 15 mmol/L, respectively, under the optimized conditions; the foam volume reached a maximum of 400 mL, and the corresponding foam’s half-life was 9.46 min The addition of SA/CL (0.1%/0.01% dosage) further significantly enhanced the LATC/AOS/HAPB mixture’s performance. The following conclusions could be obtained.

(1) The study on liquid-carrying performance demonstrated that an appropriate amount of methanol facilitated the performance of the LATC/AOS/HAPB mixture. With the presence of 50% methanol, the LATC/AOS/HAPB/SA/CL foam achieved the highest liquid-carrying efficiency of 57.44%, while the foaming efficiency remained stable at 100%.
(2) This LATC/AOS/HAPB/SA/CL mixture exhibited good tolerance to condensate oil. At the condensate oil content of up to 50%, although a slight decrease in the LATC/AOS/HAPB/SA/CL foam’s liquid-carrying ability was observed, the foaming efficiency was steady at 100% throughout the 0–55% condensate range.
(3) The system also demonstrated strong salt tolerance. Even at a high salinity of 410 g/L, the LATC/AOS/HAPB/SA/CL foam maintained a high liquid-carrying efficiency of 26.40% and a foaming efficiency of 90%, representing the highest salt resistance reported in the literature.
(4) Within the tested temperature range (30–90 °C), the liquid-carrying efficiency of the LATC/AOS/HAPB/SA/CL foam steadily increased, climbing to a peak value of 72.39% at 90 °C, while the foaming efficiency was steady at 100%.
(5) Surface tension and viscosity test results indicated significant synergistic effects within the blended system, which effectively enhance the overall performance of the LATC/AOS/HAPB/SA/CL foam. Microscopic foam morphology observations further reveal that after the addition of SA and CL, the foam liquid film thickness reaches its maximum.

## 4. Materials and Methods

### 4.1. Materials

**Figure 14:**
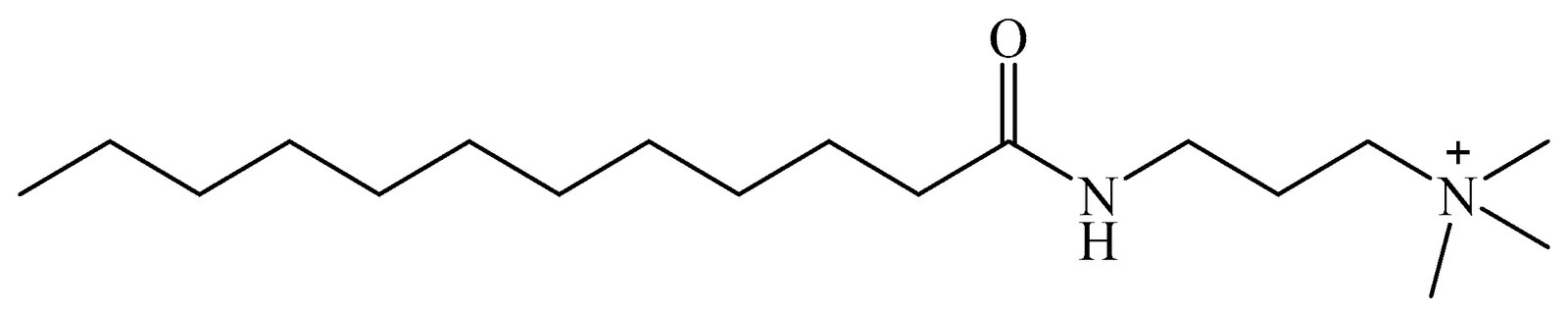
The chemical structure of LATC.

Acetonitrile (AR), petroleum ether (used as condensate, 60–90 °C boiling range), and methanol (AR) were purchased from Xi’an Kainuoke Chemical Co., Ltd. (Xi’an, China). CaCl_2_ (AR) was from Tianjin Damao Chemical Reagent Factory (Tianjin, China). Sodium alginate (SA), calcium lactate (CL), C_12_H_25_Br (99.8%) and AOS (99%) were from Shanghai Aladdin Biochemical Technology Co., Ltd. (Shanghai, China). 2-(ethylamino) ethan-1-ol was purchased from Shanghai Bide Pharmaceutical Technology Co., Ltd. (Shanghai, China). NaCl (AR), ClCH_2_COONa (98.5%), ethanol (AR), and ethyl acetate (AR) were all from Tianjin Fuyu Fine Chemical Co., Ltd. (Tianjin, China). LATC (95%), as shown in Figure 14, was acquired from Ningbo Hongbo Biotechnology Co., Ltd. (Ningbo, China). The water used in this study was distilled water produced in our lab.

### 4.2. Apparatus and Methods

#### 4.2.1. Synthesis of the Zwitterionic Surfactant HAPB

**Figure 15:**
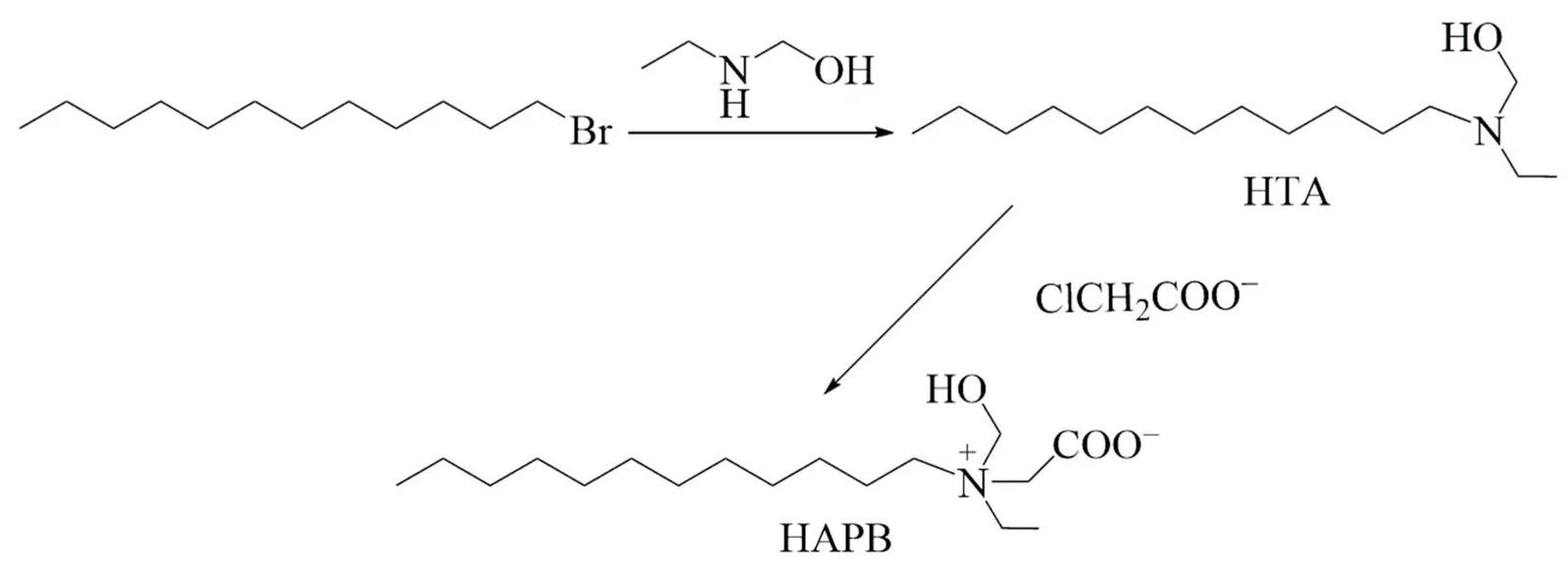
Synthesis route of HAPB.

A mixture of C_12_H_25_Br and 2-(ethylamino) ethan-1-ol (at a molar ratio of 1:1.2) was dissolved in a mixture of methanol/acetonitrile (4/1, *v*/*v*) and refluxed at 65 °C for 24 h. Then, rotary evaporation, cleaning (with petroleum ether), and drying (under vacuum conditions) were performed sequentially to produce a white solid hydroxy tertiary amine (HTA). Under an N_2_ atmosphere, the HEA/ClCH_2_COONa mixture (in a molar ratio of 1:1.2) was mixed with a certain amount of ethanol/water and then reacted under refluxing conditions at 90 °C for 36 h. The product in this step was subsequently obtained through rotary evaporation and recrystallized three times using ethyl acetate and vacuum-dried to obtain the final product, HAPB. The synthesis route is shown in Figure 15.

#### 4.2.2. Characterization of HAPB

The final product, HAPB, was characterized using a Bruker VERTEX 70 infrared spectrometer (Bruker company, Bremen, Germany).

#### 4.2.3. Foaming Capacity and Foam Stability Evaluation

For the foaming agent’s property study, each foaming agent’s foaming capacity and foam stability were inspected using a Waring blender. During measurement, 100 mL of the test solution was prepared and mixed (7000 rpm × 3 min) using a digital high-speed homogenizer (GJ-3S) to generate foams. The foam was then quickly transferred into a graduated cylinder, from which the generated foam’s volume was immediately collected as *V*_max_. Simultaneously, the time when the free liquid layer appeared in the cylinder reached half of its original volume was recorded and defined as the foam half-life, *t*_1/2_. All percentage concentrations (%) of surfactants, SA, CL, methanol and condensate in this study represented mass fractions relative to the mass of the foaming solution, unless stated otherwise. Unless specified otherwise, the foaming-related experiments were conducted at room temperature and ambient pressure conditions.

#### 4.2.4. Liquid-Carrying Performance Test

**Figure 16:**
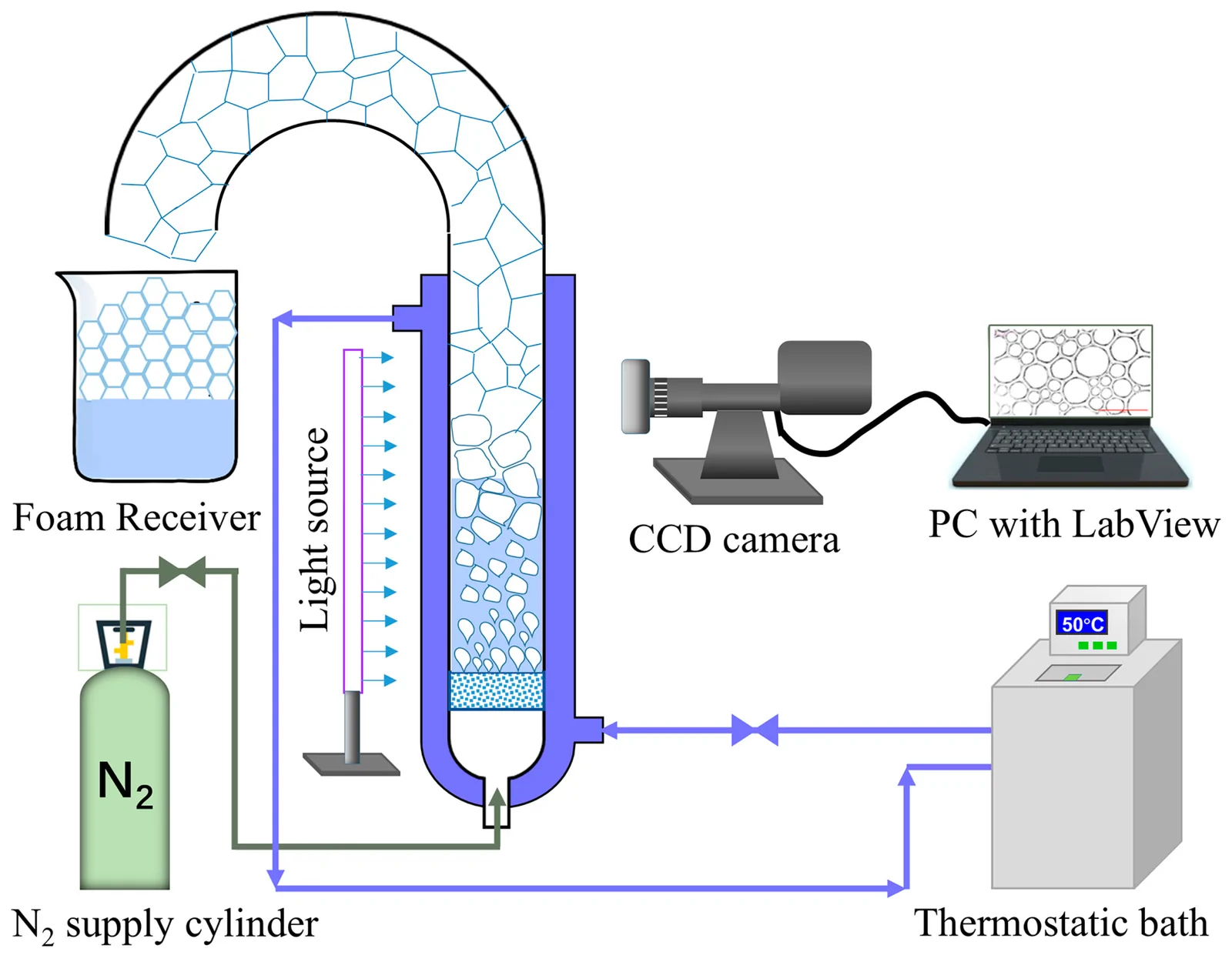
Setup for the liquid-carrying performance evaluation. The arrows linked to the light source refer to the direction of the light.

In this study, a self-assembled device, as illustrated in Figure 16, was employed to precisely record the liquid-carrying efficiency of various solutions, following the procedure provided in SY/T 7494-2020. First, 200 mL solution was pre-charged into a long transparent cylindrical tube (vertically fixed and marked with scales). An external circulating water bath temperature control device was connected to ensure the testing environment remained within a stable temperature range (from room temperature up to 90 °C). Subsequently, N_2_ (with a purity of 99%) was introduced into the column and driven upward at 400 mL/min, generating disturbance gas flow and forming foams. After continuously introducing gas for 9 min, the loaded solution in the vertically equipped column was effectively converted into a state of foams and carried out of the column. The collected foam was weighed, and the foaming solution’s liquid-carrying efficiency (*k*) and foaming ratio (*η*) were calculated following Equations (1) and (2) as listed below. To ensure data reliability, each test was independently repeated at least two times, the average value of which was adopted.

(1) $$k=\frac{m_{1}}{m_{0}}\times 100\%$$

(2) $$\eta =\frac{m_{0}-m_{r}}{m_{0}}\times 100\%$$

where *k* is the liquid-carrying efficiency, %. *η* is the foaming ratio, %. *m*_1_ is the amount of foam collected in the foam receiver, g. *m*_0_ is the amount of the preloaded solution, and g. *m*_r_ is the amount of the remaining non-foamed solution after 9 min of N_2_ injection, g.

#### 4.2.5. Surface Tension Analysis

A Sigma700 tensiometer (Biolin Scientific, Stockholm, Sweden) equipped with a Du Noüy platinum ring was employed in surface tension tests. Before each measurement, the platinum ring was cleaned with distilled water and ethanol, and then dried with pressed air. Each surface tension value was the average value of three repeated measurements.

#### 4.2.6. Viscosity Test

Each surfactant solution was sheared (1–100 s^−1^) and measured at room temperature with a HAAKE rheometer (mounted with CC41DG/Ti rotor, MARS60, Haake, Germany) to check its viscosity.

#### 4.2.7. Foam Microscopic Observation

In this study, a self-assembled setup (Figure 16) was employed to observe the microscopic morphology of foam. The entire evolution of foams generated in the column was captured with a CCD camera (VD-100, Wuxi Mhago Co. Ltd.) kept at a height of 40 cm (to the ground). During each experiment, 200 mL preloaded solution was bubbled using N_2_ gas (400 mL/min) to produce a foam with a precise height of 80 cm, and video recording was carried out immediately. After the experiment, the recorded video footage was processed using ImageJ software (version 1.52) to clearly delineate the boundaries of individual bubbles within the foam.

#### 4.2.8. Quantum Chemical Calculation

To get insight into the interactions among the surfactants used in this study, the interaction energies were calculated. The original structure of each surfactant was equilibrated at 300 K for 5 ps utilizing the gfn2-xtb method [39] with xtb software [40], during which structures were extracted subsequently every 50 fs. During equilibration, parameters were set using the default settings. The LATC/AOS and LATC/AOS/HPAB configurations were collected with the Molclus program [41]. Each collected structure was equilibrated using the gfn2-xtb method [39] in the xtb software [40]. Then each optimized structure’s energy was extracted. Electrostatic potential analysis for each optimized structure was carried out using Multiwfn software [42]. The interaction energy between two (or among three) surfactants was computed using the formula below.

(3) *E*_bind_ = *E*_complex_ − (*E*_part A_ + *E*_part B_)

The noncovalent interaction between (among) surfactants was drawn with the Independent Gradient Model (IGM) method provided in the Multiwfn software [42]. The IGM and orbitals were displayed in images with the aid of VMD [43].

## Data Availability

Data is contained within the article.
